# The pet café is a neglected site for transmission of antimicrobial-resistant Escherichia coli in urban life

**DOI:** 10.1099/mgen.0.001412

**Published:** 2025-05-23

**Authors:** Ruan-Yang Sun, Xiao-Ling Long, Ya-Li Ruan, Xi-Ran Wang, Xiao-Hui Wu, Jian Sun, Xiao-Ping Liao, Ya-Hong Liu, Hao Ren, Xin-Lei Lian

**Affiliations:** 1 National Risk Assessment Laboratory for Antimicrobial Resistance of Animal Original Bacteria, South China Agricultural University, Guangzhou, Guangdong, PR China; 2 Guangdong Provincial Key Laboratory of Veterinary Pharmaceutics Development and Safety Evaluation, South China Agricultural University, Guangzhou, Guangdong, PR China; 3 Institute of Pediatrics, Guangzhou Women and Children’s Medical Center, Guangzhou Medical University, Guangzhou, Guangdong, PR China; 4 Department of Medical Genetics, School of Basic Medical Sciences, Southern Medical University, Guangzhou, Guangdong, PR China; 5 Guangdong Laboratory for Lingnan Modern Agriculture, Guangzhou, Guangdong, PR China; 6 Jiangsu Co-Innovation Center for the Prevention and Control of Important Animal Infectious Diseases and Zoonoses, Yangzhou University, Yangzhou, Jiangsu, PR China

**Keywords:** antimicrobial resistance, *Escherichia coli*, pet café, plasmid

## Abstract

The process of urbanization has brought with it several novel lifestyles, but it remains to be seen whether such lifestyles are the potential driver behind the spread of antimicrobial resistance (AMR) in modern society. Hence, this study employs the pet café as a proof of concept to observe how one pathway of AMR transmission occurs within a megacity. A total of 111 samples were collected from consumers, workers, animals and the surrounding environment from three pet cafés in Guangzhou, and 163 bacterial strains were isolated, with *Escherichia coli* (*n*=60) being the most dominant species. The sequence type and genomic diversity of *E. coli* were observed in all three cafés. Notably, 19 highly related ST328 strains were isolated in a single pet café from both workers (skin and faeces) and animals (faeces), suggesting transmission between distinct hosts. The number of SNPs between ST328 *E. coli* isolated in this study and strains from other provinces in China was minimal, with the possibility of clonal transmission. In terms of AMR, 90% of the isolates exhibited resistance to at least three distinct classes of antimicrobials (multidrug resistance). Multiple antimicrobial resistance genes (ARGs) such as *tet*(X4) were detected in this study, and plasmid, especially hybrid plasmid, is the main transmission vector of these ARGs. Our findings highlight that the pet café is a neglected site for the transfer of ARGs among *Enterobacteriaceae*, with a propensity for continuous contamination through either clonal or horizontal transmission of ARGs.

**Impact Statement**

*Escherichia coli* is one of the most frequently isolated Gram-negative pathogens globally. It is both a widespread gut commensal of vertebrates and a versatile pathogen, with the potential to cause severe invasive disease, for which antimicrobial treatment is warranted. Consequently, the emergence of multidrug-resistant strains poses a significant challenge to effective treatment and is regarded as one of the major threats to global health. This study demonstrates the transmission of *E. coli* from shared pets to customers in the pet café, a novel urban innovation. The close interaction between shared pets and multiple batches of customers increases the risk of the transmission of multidrug-resistant (MDR) *E. coli* and antimicrobial resistance genes (ARGs), especially acquired resistance to extended-spectrum cephalosporins and tigecycline, antibiotics that are critically important in the treatment of humans. These results raise concerns about the potential transmission of ARGs to the broader community in megacities. It is, therefore, necessary to implement corresponding intervention measures to reduce the possibility of ARGs and MDR bacteria transmission.

## Data Summary

The newly sequenced genomes are listed in Table S1. These genomes have been submitted to the National Center for Biotechnology Information (NCBI), and their BioProject number is PRJNA1021388.

## Introduction

Antimicrobial resistance (AMR) is one of the top global public health and development threats. A range of factors, including antimicrobial consumption and high-density urbanization, have been identified as key drivers for AMR [1,2]. However, the full extent of the impact of emerging lifestyles and economic patterns associated with urbanization on AMR remains unclear. This knowledge gap underscores the need for further investigation to elucidate the potential effects of novel urban living and economic practices on the development and spread of antibiotic resistance. Animals, especially companion animals, are one of the important reservoirs of antimicrobial resistance genes (ARGs), posing a threat to the spread of ARGs to humans and the environment. Several studies have shown that there was a higher abundance and diversity of ARGs in the pets than in their owners, the composition of ARGs and mobile genetic element (MGE) between companion animals and their owners was found to be significantly similar and *Enterobacteriaceae* was the dominant ARG host co-occurring in the pet gut, human gut and living environment [3,4]. Nevertheless, the considerable financial implications of pet ownership deter young people from maintaining a pet in their residence. Consequently, the trend of pet café sharing animals with customers is gaining popularity in China’s major urban cities. This consumption model fulfils the human desire for interaction with animals while circumventing the high costs associated with pet ownership. These services have a public service aspect, given that animals frequently come into close contact with different individuals. Unlike pets kept at home, where owners share a stable and intimate living relationship with their pets, animals in pet cafés serve multiple batches of different customers. Unlike animals in zoos, animals in pet cafés have direct contact with customers at close quarters. Additionally, the pet café serves as a social venue, offering patrons the opportunity to engage in conversation, consume food and beverages and interact with the pets. The characteristics of pet cafés make this consumption model unique and distinct from others, potentially acting as intermediaries for the spread of antibiotic-resistant genes and leading to significant public health risks [5].

*Escherichia coli* is a diverse species of enteric bacteria that has the potential to cause severe clinical outcomes, including diarrhoea, urinary tract infections, meningitis and bloodstream infections [6]. It is well documented that *E. coli* can be either commensal or pathogenic. Due to the considerable genetic plasticity, the carriage of known pathotype-associated diarrheagenic virulence factors by *E. coli* underscores their potential to be more pathogenic [6,7]. For instance, ST131 *E. coli*, a globally dominant highly virulent multidrug-resistant (MDR) clone causing urinary tract infections and bloodstream infections, has been reported to be found in companion animals, suggesting the possibility of inter-species transmission from animals to their owners [8,9]. Additionally, *E. coli* is regarded as a significant reservoir for multiple clinically important ARGs and an excellent indicator of AMR transmission between distinct hosts [10,11]. The first reports of multiple clinically important ARGs, including *mcr-1* and *tet*(X4), were published in the context of *E. coli* [12,13]. Often, the horizontal gene transfer of ARGs between bacteria was facilitated by MGEs, such as transposons, integrons and plasmids [14]. Various plasmid types such as IncF and IncHI2 plasmids have been extensively documented as vectors for the horizontal spread of ARGs, playing an important role in ARG transmission [15,16]. The co-occurrence of virulence genes and ARGs could contribute to the higher mortality of immunocompromised patients with *E. coli* bacteremia [17], representing a significant public health threat.

In this study, we compared the phylogenetic relationship and the ARG profile of *E. coli* collected from pets, humans and surfaces in the café environments and explored the potential inter-host transmission between pets and customers. In summary, our findings elucidate the genomic structure and population diversity of *E. coli* from pet cafés in a megacity to understand the potential impact of close contact between customers and shared pets.

## Methods

### General characteristics of pet cafés

Three pet cafés were selected for sample collection in this study. Two cafés were located in Tianhe district, the other one was in Yuexiu district and each café was located over 7 km away from the other two cafés. They exhibited homogeneous spatial configurations and operational protocols. All three investigated pet cafés were indoor confined environments with limited floor area, each equipped with sanitation facilities (restrooms/handwashing stations). There are more washing stations in café 3 than in other cafés. While designated zones were allocated for different animal species, free-roaming access throughout the pet cafés was permitted. Faeces from animals were not promptly removed according to standardized hygienic protocols. Customers were permitted to engage in direct interactive activities including tactile contact with animals and the provision of feed. Furthermore, these pet cafés provided beverage and food services, enabling customers to eat food while engaging in animal interactions.

### Sample collection

From January 2022 to June 2022, a total of 111 samples of animal faeces (48 samples), human faeces (2 samples) and skin (including customers and workers, total 14 samples), animal skin (13 samples) and surrounding environment (34 samples) were collected from three pet cafés in Guangzhou, Guangdong, China. Collection protocols were listed as follows: (1) animal faecal sample collection – fresh pet faeces were collected using sterile cotton swabs and transferred into 50-ml centrifuge tubes. (2) Human faecal sample collection: fresh human stool specimens were obtained using sterile collection bags. (3) Human skin sample collection: sterile saline-moistened cotton swabs were used to swab human hand and head surfaces, with subsequent placement of swabs into centrifuge tubes containing 2 ml of sterilized saline. (4) Animal skin sample collection: epidermal and fur samples from animals were swabbed using sterile cotton swabs moistened with saline solution. The swabs were then placed in centrifuge tubes containing 2 ml of sterilized saline. (5) Environmental sample collection: interaction tools, handwashing sinks, cages and other surfaces in pet cafés were sampled using sterile saline-moistened cotton swabs. (6) Pet drinking water collection: drinking water samples were directly collected from water dispensers into 50-ml sterile centrifuge tubes. Collected swabs were deposited into centrifuge tubes containing 2 ml of sterilized saline. All samples were collected, set into sterile sacks and stored at temperatures underneath 4 ℃.

### Bacterial isolation

The samples were transported promptly to the laboratory for further examination (Table S1, available in the online Supplementary Material). The details of bacterial isolation are conducted as follows: (1) animal and human faecal – a faecal aliquot was aseptically transferred from the collection tube into a 2-ml sterile centrifuge tube containing 1 ml of normal saline (0.9% NaCl). The mixture was vortex-mixed thoroughly for homogeneous suspension. Subsequently, 50 µl aliquots of the homogenized faecal suspension were aspirated and evenly spread onto MacConkey agar plates using a sterile spreading rod. (2) Animal/human skin and environmental samples: collected samples were directly subjected to vortex homogenization. Following homogenization, 50 µl aliquots of each sample suspension was aseptically transferred and uniformly inoculated onto MacConkey agar plates using a sterile spreading rod. (3) Pet drinking water: a 50 µl aliquot was directly aspirated from the water sample and streaked evenly onto MacConkey agar plates employing a sterile spreading rod. Diluted samples were incubated for 18 h at 37 °C. All colonies from each sample that were bright peach or reddish with a dark peach centre were selected.

### Genome sequencing and bioinformatics analysis

The genomic DNA of all isolates was extracted using the TIANamp Bacteria DNA Kit (Tiangen, Beijing, China) and subjected to 250 bp paired-end whole-genome sequencing (WGS) using the Illumina HiSeq system (San Diego, CA, USA). Trimmed reads were assembled by SPAdes v3.6.2 [18]. Multilocus sequencing typing (MLST) and phylogenetic groups of *E. coli* were analysed using the mlst tool (https://github.com/tseemann/mlst/) and the ClermonTyping method [19], respectively. A minimum spanning tree of all STs was generated by Phyloviz v2.0 using the BURST algorithm [20]. AMR genes, target mutations in QRDRs, virulence factors and plasmid replicon types were obtained using AMRFinder v3.8.4 [21] and ABRicate v1.0.1 (https://github.com/tseemann/abricate). The network graph describing the co-occurrence of ARGs and plasmid replicons was constructed using Gephi v0.9.2 [22], and principal component analysis (PCA) was measured using the ‘ggord’ package. MOB-suite with MOB-recon and MOB-typer software modules was used to partially reconstruct and type the plasmids from short-read genome assemblies [23].

### Antimicrobial susceptibility testing

The minimum inhibitory concentrations (MICs) of 18 antimicrobial agents for *E. coli* isolates were determined using the agar dilution method, and the results were interpreted according to the European Committee on Antimicrobial Susceptibility Testing (v10.0, for tigecycline and colistin) and the Clinical and Laboratory Standards Institute document (M100-S28 and VET01-S2, for the remaining antibiotics) [24,25]. *E. coli* ATCC 25922 served as the quality control strain.

### Phylogenetic analysis

All *E. coli* assemblies were used for core-genome alignments to construct phylogenetic trees using Snippy v4.6.0 (https://github.com/tseemann/snippy). SNPs were called, and recombinant regions were removed using Gubbins v2.4.1 and the final SNPs extracted with SNP-sites [26,27]. Maximum likelihood (ML) phylogenetic trees were inferred using RAxML v8.2.12 (GTRGAMMA substitution model) [28]. The final tree was mid-point rooted and visualized with iTOL v5 [29]. Pairwise SNPs between strains were counted from the core SNP alignment with snp-dists v0.7.0. The lineages of the phylogenetic tree were defined using rhierBAPs [30].

To provide a global context, additional ST328 *E. coli* genomes were downloaded from the National Center for Biotechnology Information (NCBI) Pathogen database (https://www.ncbi.nlm.nih.gov/pathogens/) (access date: 15 July 2024). Basic information of all genomes was collected from the NCBI by using an automated python script. A core-genome non-recombinant ML tree was constructed and visualized, as described above.

### Pan-genome analysis

For pan-genome analysis, all *E. coli* genomes were annotated by Prokka v1.14.6 [31], and annotation files were carried out using Panaroo v1.3.3 to cluster the genes encoding complete protein sequences into core (core and soft core) and accessory (shell and cloud) genomes [32]. We chose a percentage identity of 95% to distinguish between the core genes and the accessory genes. Non-metric multidimensional scaling (NMDS) was performed to calculate the similarity coefficient by Jaccard Index at ARG level, virulence factor level and pan-genome level using the vegan R packages [33].

### Long-read sequencing of a representative *E. coli* strain

Five representative isolates were selected for long-read sequencing using the ONT MinION Platform (Nanopore, Oxford, UK). Isolates were selected for sequencing based on the diversity of AMR profiles and lineage membership. ONT sequencing was performed using the R9.4 flow cell (FLO-MIN106). Raw reads generated by MinKNOW were basecalled and barcoded using Guppy. The *de novo* hybrid assembly of both short (Illumina) and long reads (ONT) was performed using Unicycler v0.5.0 [34]. Mobility prediction was recognized using MOB-suite v 3.1.9 [23]. Sequence comparison of chromosomes and plasmids was conducted using Clinker v0.0.25 and BRIG [35,36].

## Results

### Bacterial isolation and antimicrobial resistance profile

**Fig. 1.**
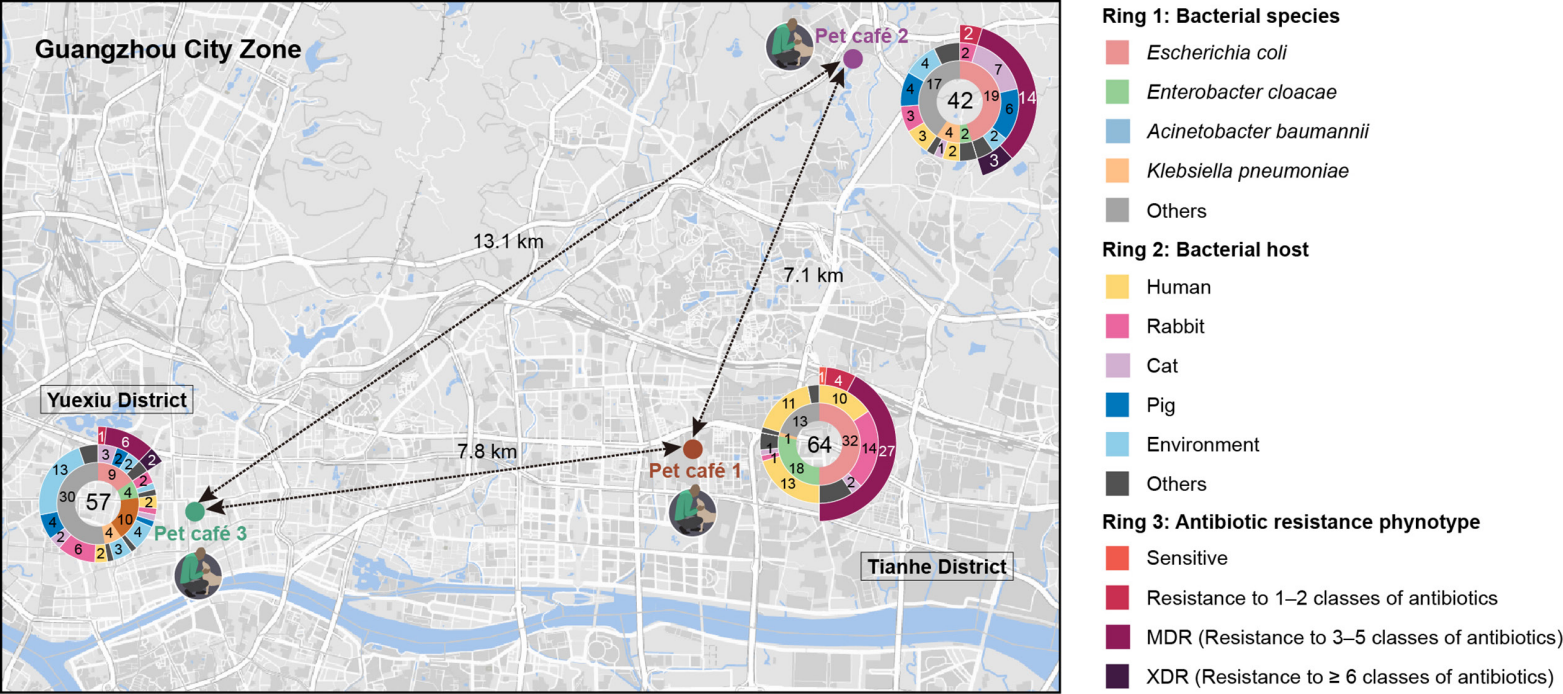
Geographical distribution of sampled points in the region of Guangzhou city. The locations of three sampled pet cafés are indicated by circled icons, and the distance between each point is indicated. The total number of bacteria collected from each sampling location is indicated in the centre of the sunburn diagrams. The inner ring represents the proportion of different species, including *E. coli*, *Enterobacter cloacae*, *A. baumannii*, *K. pneumoniae* and others. The middle ring represents the distribution of different bacterial species across a range of hosts in each sampling pet café. The outer ring represents the number of *E. coli* MDR phenotype.

Overall, a total of 163 Gram-negative bacteria were obtained by growing the collected samples in MacConkey media. All strains were subjected to WGS, and the combined species identification indicated that 163 isolates belonged to eight *Enterobacteriaceae* species, including *E. coli* (*n*=60), *Enterobacter cloacae* (*n*=25), *Acinetobacter baumannii* (*n*=10), *Klebsiella pneumoniae* (*n*=9) and other species (Fig. 1 and Table S1). This demonstrates a high bacterial diversity over pet café niches. The predominant species among the entire strain collection is *E. coli*, which was detected in all three pet cafés. Accordingly, subsequent analysis will concentrate on *E. coli* strains.

Antimicrobial susceptibility tests of the 60 *E. coli* strains revealed that these isolates exhibited high resistance to most of the antibiotics tested. Of these strains, 52 were classified as MDR (defined as resistance to more than three categories of antibiotics). The majority of *E. coli* strains were resistant to tetracycline (91.7%), sulfamethoxazole/trimethoprim (78.3%), nalidixic acid (63.3%) and ampicillin (55.0%). The resistance rates were found to be lower for florfenicol (33.3%), gentamicin (28.3%), chloramphenicol (25.0%), cefotaxime (11.7%), ceftazidime (10.0%), ceftriaxone (10.0%), ciprofloxacin (10.0%), aztreonam (6.7%), colistin (5.0%) and tigecycline (1.7%). All isolates were susceptible to amikacin, meropenem and fosfomycin (Fig. S1 and Table S2).

**Fig. 2.**
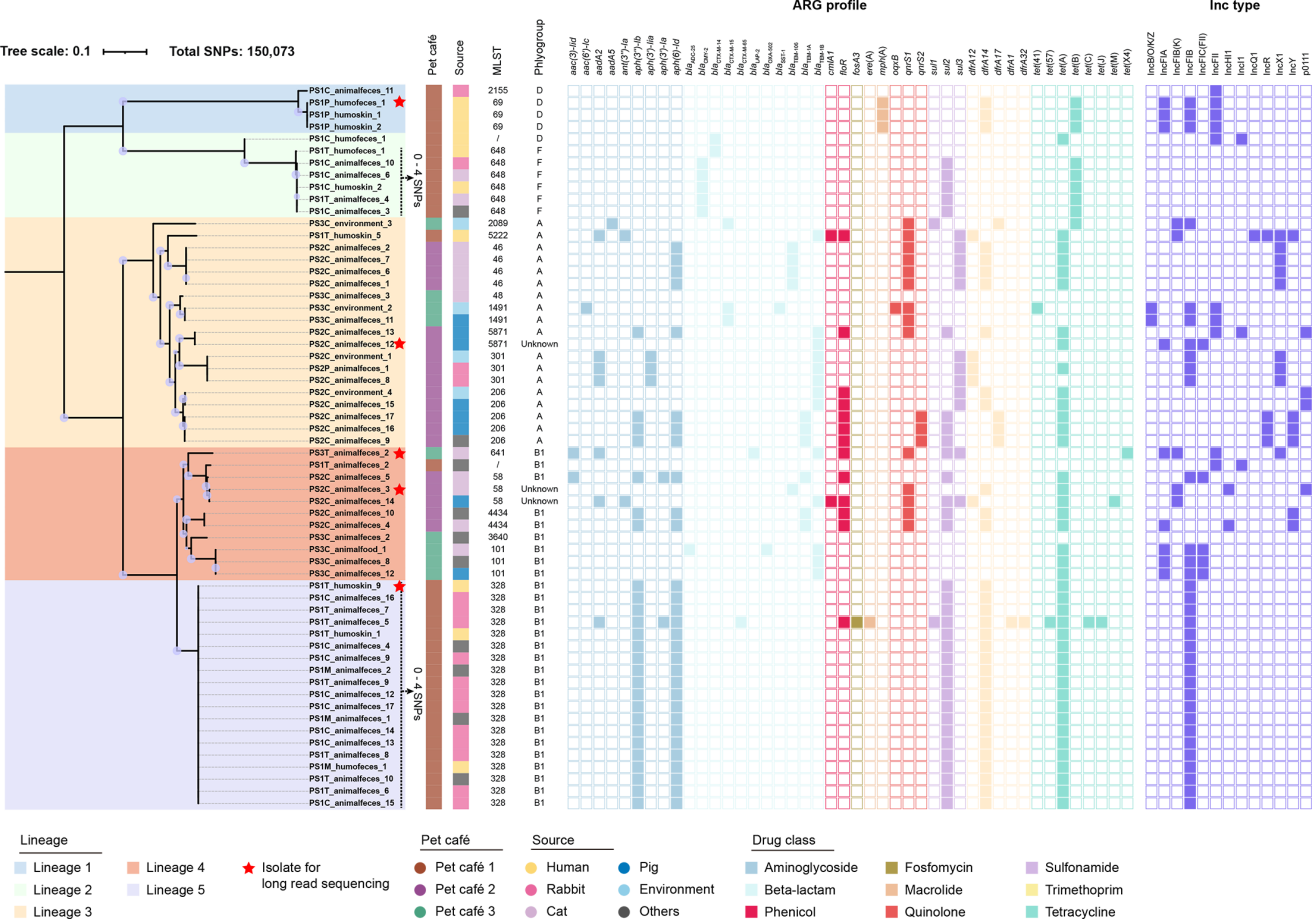
Genomic analysis of 60 *E. coli* isolates in three pet cafés in Guangzhou city. An ML phylogenetic tree was constructed using the core-genome SNPs and midpoint rooted. Internal nodes are labelled with circles indicating branch >70% bootstrap support. The shadow represents the clade, and the rectangular colour blocks represent three different pet cafés. The presence or absence of different classes of antibiotic resistance genes and plasmid replicons is denoted by filled and empty squares, respectively.

**Fig. 3.**
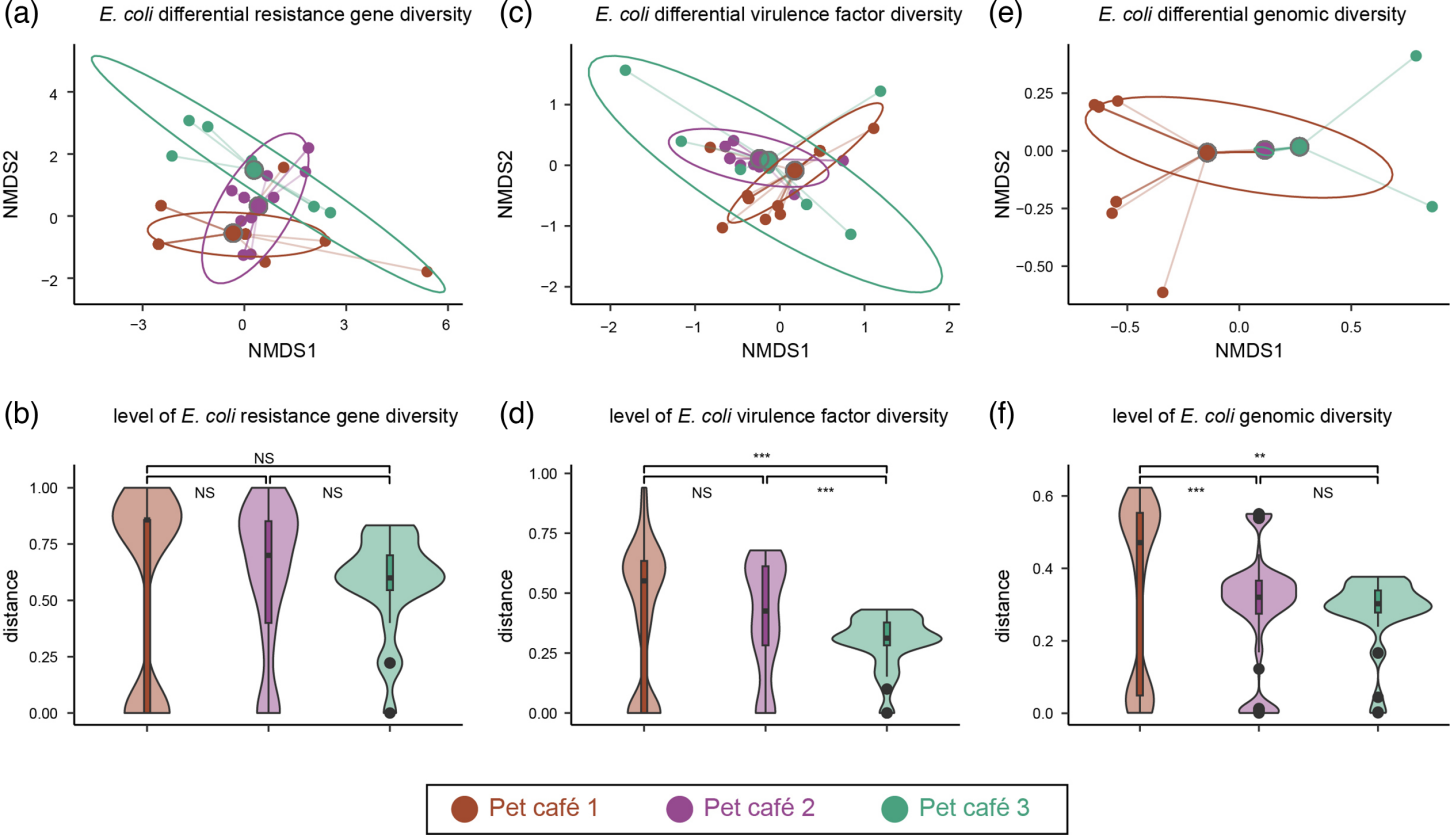
NMDS and violin plots according to Jaccard distance index and the pet café (indicated by colours). (**a**) Differential antibiotic resistance gene content. (**b**) Level of antibiotic resistance gene diversity. (**c**) Differential virulence factor content. (**d**) Level of virulence factor diversity. (**e**) Differential genomic content based on pan-genome analysis. (**f**) Level of genomic diversity based on pan-genome analysis. Highly significant differences are indicated by *** (*P*<0.001). Significant differences are indicated by ** (*P*<0.01). Non-significant differences are indicated by ns.

For genotype, 44 acquired antimicrobial resistance genes were identified in the 60 isolates tested. A median of 5 ARGs (range 0–17) was detected in the genome of each strain. Genes coding for resistance to tetracyclines [*tet*(A), 41/60], sulfonamides (*sul2*, 34/60; *sul3*, 12/60), aminoglycosides (*strA*, 27/60; *strB*, 31/60) and trimethoprims (*dfrA14*, 33/60) were highly present. Plasmid-mediated quinolone resistance genes (*qnrS1*, 15/60; *qnrS2*, 3/60; *oqxB*, 1/60), third-generation cephalosporins (*bla*_CTX-M-15_, 3/60; *bla*_CTX-M-14_, 2/60; *bla*_CTX-M-65_, 1/60) and phenicols (*floR*, 14/60) were also detected in the isolates. Surprisingly, the tigecycline resistance gene *tet*(X4) was detected in one of the 60 *E. coli* isolates (Fig. 2). The AMR genotype was a good predictor of phenotypic MDR and XDR with a high concordance between genotype and phenotype. The contribution of each combination of resistance genes to the MIC of an antimicrobial compound is shown in Fig. S2. The *E. coli* isolates were also found to possess numerous virulence-associated factors, including adherence, effector delivery system, invasion and regulation (Fig. S3). The *E. coli* populations from all three pet cafés exhibited a notable divergence in the antibiotic resistance gene and virulence factor content (Fig. 3a, c), showing distinct resistance mechanisms to particular antimicrobial classes. However, the sum of genetic resistance determinants carried by *E. coli* within each pet café demonstrated no statistically significant difference between each other (Fig. 3b). Furthermore, a significantly higher diversity was observed in the virulence factor from pet cafés 1 and 2 when compared to pet café 3 (Fig. 3d).

### Genomic diversity of *E. coli* in pet cafés

*In silico* MLST clustered the 60 *E. coli* into 17 distinct sequence types (STs) and 2 unknown STs, with pet café 1 clustered in 5 STs and pet café 2 and café 3 clustered in 6 STs (Table S1). MLST data for the 60 isolates were used to generate a minimum spanning tree. Distinct STs were associated with the different pet cafés, with a high level of genetic diversity observed overall (Fig. S4). ST328 was the dominant sequence type, accounting for 31.7% (*n*=19), followed by ST648 (*n*=6) and ST206 (*n*=5). ST328 isolates were detected in the same pet café and were associated with isolates from worker skin (*n*=2), worker faeces (*n*=1), rabbit faeces (*n*=12) and other animal (deer, hedgehog and alpaca) faeces (*n*=4), suggesting this particular pet café is the common source of ST328 *E. coli*, and continued contamination of humans and pets occurred. The *E. coli* isolates were classified into four groups using Clermont typing, with the majority of isolates belonging to groups B1 (*n*=27, 45.0%) and A (*n*=18, 30.0%). These two groups are typically associated with commensal strains [37]. The combined MLST and Clermont typing analysis revealed pronounced genetic diversity among the *E. coli* isolates tested in this study.

To better understand the genetic diversity of the *E. coli* in the pet café, an ML phylogeny was constructed based on 150,073 core-genome SNPs (Fig. 2). Five sequence clusters (SCs) were classified by hierBAPs analysis. Lineage 5 encompassed 19 isolates from the same pet café, exhibiting a high degree of whole-genome sequence similarity (pairwise SNPs≤4) between workers’ skin, workers’ faeces and shared pets’ faeces (Table S3). Similarly, the ST648 isolates obtained from the skin of workers and the faeces of pets (cats, rabbits and dogs) were identified as belonging to lineage 2 and exhibited four distinct SNPs. This indicates that they originated from a common ancestor and provides evidence of direct transmission of *E. coli* among humans and pets within one pet café. Lineage 3 and lineage 4 comprised isolates from all three cafés. Based on the phylogenetic tree and diversity of MLST types analysis, *E. coli* strains isolated from pet and human faeces and environmental samples in three pet cafés were diverse and exhibited no obvious clonal spread between pet cafés in one megacity. Pan-genome analysis based on the 60 isolates showed that a total of 18,909 genes were identified, of which 16,726 were assigned to the accessory genome. The accessory genes accounted for 88.5%, encompassing a remarkable diversity and high plasticity among these strains. Unlike the lower diversity of ARG content observed between each café, the genomic diversity of *E. coli* was found to be statistically significant. The diversity was higher in the total *E. coli* population from pet café 1 compared to the *E. coli* population from pet café 2 and pet café 3, considering the genes that were present and absent in each café from the total pan-genome (Fig. 3e, f).

**Fig. 4.**
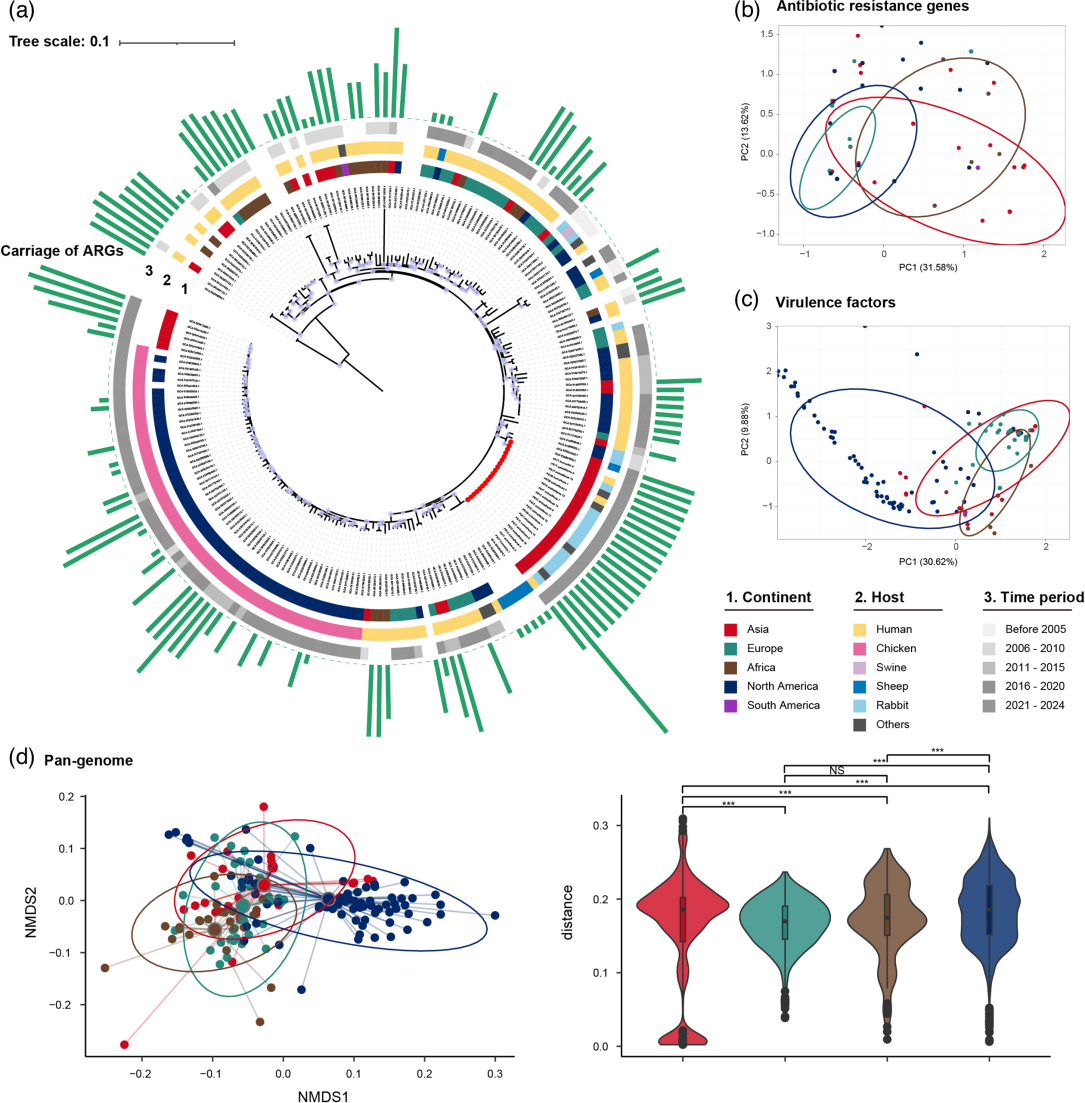
Phylogenetic structures of Chinese ST328 *Escherichia coli* in a global context. (**a**) ML tree outlining the phylogenetic structure of 19 ST328 *E. coli* isolates unique to this study (highlighted by the red points) combined with 190 global ST328 *E. coli* isolates. Internal nodes are labelled with circles indicating branch >70% bootstrap support. Metadata are visualized as follows from inner to outer: 1, geographic area of origin; 2, host of isolation; 3, year of isolation; and 4, number of ARGs carriage. (**b**) PCA for antibiotic resistance genes in the five continents. (**c**) PCA for virulence factors in the five continents. (**d**) NMDS and violin plots according to the Jaccard distance index based on ST328 *E. coli* pan-genome analysis. Highly significant differences are indicated by *** (*P*<0.001). Non-significant differences are indicated by ns.

To provide further contextualization of our data, we conducted a phylogenetic comparison of 19 ST328 isolates from our study with 190 publicly available ST328 sequences (Table S4). ST328 *E. coli* were mainly distributed in the USA (*n*=61, 29.2%), the UK (*n*=27, 12.9%) and China (*n*=23, 11.0%). The ST328 strains were observed to be distributed in the clinic (*n*=83, 39.7%) and chicken (*n*=57, 27.3%). All ST328 strains belong to phylogroup B1 and carry the *eae* gene, identified as enteropathogenic *E. coli* (EPEC). The phylogenetic analysis revealed a high degree of diversity, with multiple small clades (Fig. 4a and Table S5). A sheep strain from Jiangsu province, China, in 2019 and another strain from Zhejiang province, China, in the same year had high similarity (133–145 SNPs) with the cluster of 19 isolates collected in this study, clustering in the same branch. There was a possibility of transmission between different hosts, indicating that a specific ST328 clone has been circulating for 4 years in China and is phylogenetically distinct from European or American isolates. In addition, the number of ARGs carried by this specific type of ST remained high, with a range of 0 to 19 (Table S4). A similarity analysis of ARG and VF composition among ST328 strains was performed using PCA. Although the composition of ARGs was found to be similar between samples from five continents, the strains from Europe were found to be closely clustered, and strains from other continents possessed a level of variance (Fig. 4b). Moreover, the composition of VFs in strains from Europe and Africa was closely clustered, whereas strains from Asia and North America exhibited evident scattering (Fig. 4c). Based on the pan-genome analysis, ST328 *E. coli* comprised 11,395 unique genes, with accessory genes (present in less than 99% of the isolates) accounting for 69.2%, covering a considerable ST328 *E. coli* diversity. NMDS was performed based on the accessory gene content matrix to investigate genetic composition differences between each continent (Fig. 4d). The genes comprising the pan-genomes of ST328 *E. coli* from different continents were found to be statistically different, except between Europe and Africa, indicating that this specific ST type has undergone a localized expansion in each continent.

### Genetic analysis of MDR strains

**Fig. 5.**
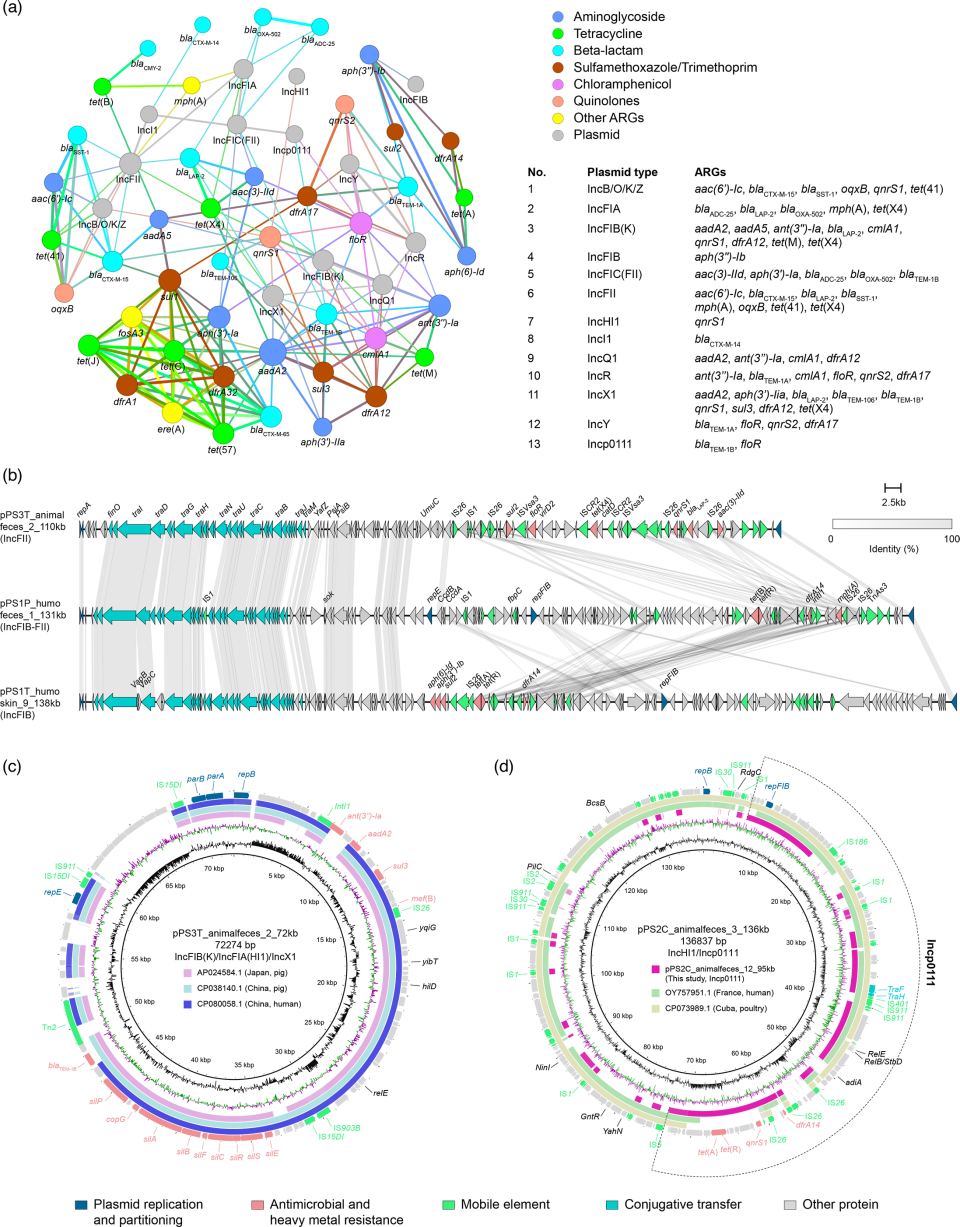
Genetic basis of multiple ARGs in this study. (**a**) Co-occurrence pattern of ARGs with plasmid replicons. The nodes represent ARGs or plasmid replicons. Connections between nodes with the same colour indicate that they are related. Moreover, the line’s thickness is proportional to the correlation strength, with a thicker line indicating a stronger relationship between the variables. All associated genes in the figure had *P* values less than 0.05. The co-occurrence between ARGs and plasmid replicons is annotated on the bottom right. (**b**) The linear alignment of complete IncF plasmids in this study. (**c**) The circular genetic map of the IncFIB(K)/IncFIA(HI1)/IncX1 plasmid in one animal faeces isolate and other plasmids deposited in the GenBank database. (**d**) The circular genetic map of the IncHI1B/Incp0111 and Incp0111 plasmids from animal faeces and other plasmids deposited in the GenBank database.

To explore the co-relationship between ARGs and plasmid replicons, a correlation analysis was constructed. When the Spearman value > 0.3 and *P* < 0.05, it was hypothesized that the non-random co-occurrence patterns between ARGs and plasmid replicon could indicate the possible vector information of ARGs [38]. The visualization of the network consisted of 57 nodes and 334 edges, and the modularity index of the network was 0.621 (values>0.4). Twelve types of plasmids were speculated as the possible ARG vectors based on the co-occurrence results. For instance, *tet*(X4) was strongly associated with the IncF plasmid. Similarly, *bla*_CTX-M-14_ was strongly associated with IncI1 plasmid (Fig. 5a). Using MOB-suite, we were able to partially reconstruct and characterize the 117 plasmids carrying known replicons for 54 draft genome assemblies of overall 60 isolates. Eighty-three (70.9%) of them were predicted as conjugative or mobilizable (Table S8).

The utilization of long- and short-read hybrid assembly enabled the comprehensive elucidation of the genomic structure of five representative *E. coli* strains, selected based on their origin, ST, antibiotic resistance gene composition and plasmid content (Fig. 2). We found that no ARGs are located on the chromosome in the five complete sequenced isolates, plasmids being the main vector of transmission of these ARGs (Table S9). Based on the MOB-suite, all complete plasmids were predicted to be conjugative or mobilizable. IncFII plasmid is the most prevalent plasmid type among the strains and possesses typical and conserved IncF backbones such as replication genes and transfer genes. However, the variable region is highly diverse. Multiple insertion sequences such as IS*26* and IS*CR2* contribute to the horizontal transfer of ARGs. It is important to note that the *catD* hydrolase with *tet*(X4) is flanked by a complete and a truncated copy of IS*CR2* in the same orientation, which are inserted into the MDR region (Fig. 5b). IS*CR2* has previously been reported to be associated with the generation of the circular form, facilitating the mobilization of *tet*(X4), mediating resistance to last resort antibiotic tigecycline [39].

Plasmids in pet cafés also possessed high genetic plasticity. Hybrid and multi-replicon plasmids were detected, and that was likely generated by recombination between different plasmid backbones. The presence of multiple replicons in pet cafés might prevent plasmid incompatibility and facilitate their interaction with a broad range of hosts [37]. Plasmid pPS3T_animalfaeces_2_72 kb was identified as an IncFIB(K)/IncFIA(HI1)/IncX1 hybrid plasmid. The sequence organization was found to be similar to that of plasmid p2-Ec387 (AP024584.1, Japan, pig, coverage 76%, identity 99%), plasmid pG3X16-2-3 (CP038140.1, China, pig, coverage 80%, identity 99%) and plasmid p655Rt (CP080058.1, China, human, coverage 76%, identity 99%) (Fig. 5c). Plasmid pPS2C_animalfaeces_3_136 kb was likely formed by fusion between the IncHI1 plasmid and an Incp0111 plasmid. blastn comparison of the IncHI1/Incp0111 hybrid plasmid with those in the NCBI database showed more than 99% nucleotide identity at 82% coverage to two other plasmids, namely pF1053-2 (OY757951.1, France, human) and pYLMB10a (CP073989.1, Cuba, poultry) (Fig. 5d). Interestingly, we also identified an Incp0111 plasmid (pPS2C_animalfaeces_12_95 kb) in the same café, two plasmids (pPS2C_animalfaeces_3_136 kb and pPS2C_animalfaeces_12_95 kb) isolated from different animals (cat and pig) in one café shared 99% nucleotide identity and 45% coverage, and IS*5* served as a potential recombination site for plasmid recombination, suggesting the potential for the formation of cointegrate plasmids and continued transmission in one café (Fig. 5d).

## Discussion

In the present study, we aimed to investigate the antimicrobial traits and genetic characteristics of *E. coli* isolates obtained from pet cafés in Guangzhou, the third megacity in China. Of the multiple bacterial species identified, *E. coli* was the most frequently isolated in the majority of samples. Whole-genome sequencing analysis revealed that 60 *E. coli* from different pet cafés belonged to 17 different ST types. Luckily, many known to cause extraintestinal infection STs of *E. coli* such as ST131, ST10 and ST38 were not detected [40,42]. Among STs, ST328 was the most prevalent type, collected from customers, workers, animal faeces and surrounding environments within a single pet café. Phylogenetic analysis revealed a high possibility of transmission between workers and shared pets in the same café. Pairwise SNP analysis suggested that one ST328 *E. coli* isolate derived from worker faeces (PS1M_humofaeces_1) and the other ten ST328 *E. coli* isolates derived from pet faeces (rabbits and pigs) belonged to a single clone (SNP=0). It is important to note that rabbits are coprophagic, so they are likely to share faecal flora if they are co-housed. Furthermore, the contamination of the skin may be transient and not result in faecal contamination unless handwashing is performed. The detection of similar strains in both the skin and faeces of workers suggests that prolonged contact with animals without adequate disinfection may result in the colonization of the human body. These results showed the high clonality of the strains circulating within the same pet café, underlying that pet cafés in megacities may represent an unexpected site for ARG spillover due to direct contacts between animals and humans, by which the arb and ARGs are expected to transmit frequently [43]. ST328 is an underreported sequence type, and only a few ST328 *E. coli* isolates have been identified in the global collection. The positive rate of the virulence factor *eae* was 100% in ST328 strains, all belonging to EPEC. A similar phenomenon was obtained in ST328 strains collected from rabbits in Sichuan, China [44]. However, the results of this study showed that these ST328 *E. coli* isolates were all MDR isolates. It is worth noting that only a small number of SNPs differed between the strains isolated from humans and pets in Guangdong in this study and *E. coli* from other Chinese provinces, and there was a possibility of transmission between different hosts in China. It is important to note that the main mode of transmission for ARGs and their hosts in the environment is through direct and indirect contact, such as air and surfaces, and contaminated food and water [45,46]. The exchange of ARGs and their hosting bacteria occurred in the pet café. The shared pets have close contact with numerous batches of customers from different areas in the city throughout the day, often without any disinfection measures in place until the café closes. This consumer pattern increases the likelihood of shared pets serving as carriers for the spread of ARGs and also presents a potential for sustained transmission.

*E. coli* isolates from various pet cafés exhibited distinct genetic compositions, while the level of ARG diversity was similar across all pet cafés, revealing that horizontal gene transfer is the primary mechanism for ARGs transmission. The pattern of co-occurrence between ARGs and mobile elements revealed by the network analysis helps to better explain the correlation between them [38]. The network analysis results showed that IncF plasmids were strongly associated with *tet*(X4), *qnrS1*, *oqxB*, *bla*_CTX-M-15_ and *bla*_OXA-502_ (R>0.3, *P*<0.05). This phenomenon suggests that plasmids such as IncF (especially IncFII) plasmids play a critical role in facilitating the spread of ARGs [47,48]. Tigecycline, one of the few antibiotics used as a last resort, was approved by the FDA in 2005 for the treatment of complicated skin and skin-structure infections as well as complicated intra-abdominal infections [13]. Tigecycline is not licensed for veterinary use. However, the production and use of tetracyclines are highest among all antimicrobial compounds in China [49]. One isolate containing *tet*(X4) located on an IncFII plasmid was detected in animal faeces in one café. The core genes including replicon, maintenance and transfer gene cluster were detected in the IncF plasmids, indicating the possibility of transfer of this clinically important ARG from pets to the customers in one café. In addition, different types of plasmids were also identified in this study, and most of them are hybrid plasmids. Hybrid plasmids are now commonly observed and possess high genetic plasticity, characterized by an MDR mosaic region and a hybrid backbone likely formed through recombination between different plasmid backbones [50,52]. These recombination events may contribute to expanding the plasmid host range through the integration of multiple replicons and enable adaptation to environmental pressures through the acquisition of ARGs and heavy metal tolerance genes.

We acknowledge that our study has several limitations. Firstly, our investigation was conducted on a limited scale, involving only three pet cafés in Guangzhou to examine the persistence of *Enterobacteriaceae*. Secondly, detailed information regarding the origin of the pets in the café and their medication history was lacking. Thirdly, no systematic evaluation of the efficacy of cleaning and disinfection of the pet cafés during the study period was conducted in this study. Therefore, further research is imperative to investigate the potential association between the persistence and transmission of *Enterobacteriaceae* and the effectiveness of cleaning and disinfection practices in these environments.

## Conclusion

In summary, we have described the genetic landscape and relationship of ARGs between shared pets, workers and customers in pet cafés in China. Moreover, the present study demonstrates that the *E. coli* from multiple distinct origins within the same café exhibit a close phylogenetic relationship. Moreover, plasmids, particularly hybrid plasmids, facilitate the transmission of ARGs, including *tet*(X4) and others. Overall, in this study, we provided a novel insight into the pet café, a neglected site for enrichment and transmission of antimicrobial-resistant *E. coli* in urban life. This novel urban lifestyle may serve as a medium of continuous contamination by either clonal or horizontal transmission of ARGs. This study highlights the potential risk that pet cafés pose for the transfer of ARGs among *Enterobacteriaceae* and represents a previously underestimated threat from potential health hazards.

## Supplementary material

10.1099/mgen.0.001412 Uncited Supplementary Material 1.

10.1099/mgen.0.001412 Uncited Supplementary Material 2.

## Abbreviations

AMR: antimicrobial resistance
ARGs: antimicrobial resistance genes
EPEC: enteropathogenic *Escherichia coli*
MDR: multidrug-resistant
MGE: mobile genetic elements
MIC: minimum inhibitory concentration
ML: maximum likelihood
MLST: multilocus sequencing typing
NMDS: non-metric multidimensional scaling
PCA: principal component analysis
SCs: sequence clusters
STs: sequence types
