# Supplementary material for: The pet café is a neglected site for transmission of antimicrobial-resistant Escherichia coli in urban life
Source: Microb Genom. 2025 May 23;11(5):001412. doi: 10.1099/mgen.0.001412 (PMC12102497; doi:10.1099/mgen.0.001412)
Supplement: Uncited Supplementary Material 1. [file mgen-11-01412-s001.pdf]

Supplementary Materials for

The pet café is a neglected site for transmission of antimicrobial-resistant *Escherichia coli* in urban life

Ruan-Yang Sun[1,2#], Xiao-Ling Long[1,2#], Ya-Li Ruan[1,2], Xi-Ran Wang[1,2], Xiao-Hui Wu[3,4], Jian Sun[1,2,5], Xiao-Ping Liao[1,2,5], Ya-Hong Liu[1,2,5,6], Hao Ren[1,2,5*], Xin-Lei Lian[1,2,5*]

[1]National Risk Assessment Laboratory for Antimicrobial Resistance of Animal Original Bacteria, South China Agricultural University, Guangzhou, Guangdong, P. R. China.

[2]Guangdong Provincial Key Laboratory of Veterinary Pharmaceutics Development and Safety Evaluation, South China Agricultural University, Guangzhou, Guangdong, P. R. China.

[3]Institute of Pediatrics, Guangzhou Women and Children's Medical Center, Guangzhou Medical University, Guangzhou, Guangdong, P. R. China.

[4]Department of Medical Genetics, School of Basic Medical Sciences, Southern Medical University, Guangzhou, Guangdong, P. R. China.

[5]Guangdong Laboratory for Lingnan Modern Agriculture, Guangzhou, Guangdong, P. R. China.

[6]Jiangsu Co-Innovation Center for the Prevention and Control of Important Animal Infectious Diseases and Zoonoses, Yangzhou University, Yangzhou, Jiangsu, P. R. China.

[#] These authors contributed equally.

[*] Corresponding Author: Hao Ren, Email: hao.ren@scau.edu.cn; Xin-Lei Lian, E-mail: xinlei_lian@scau.edu.cn.

Supplemental Tables

Table S1. Metadata of 163 strains sequenced in this study

Table S2. Antimicrobial susceptibility testing of 60 *Escherichia coli* and 9 *Klebsiella pneumoniae* strains (MICs, mg/L)

Table S3. ARG genotype of 60 *Escherichia coli* strains

Table S4. Virulence factor genotype of 60 *Escherichia coli* strains

Table S5. Pairwise comparison of single nucleotide polymorphisms of 60 *Escherichia coli* isolates

Table S6. Information regarding of 190 ST328 *Escherichia coli* strains obtained from the NCBI database

Table S7. Pairwise comparison of single nucleotide polymorphisms of 209 ST328 *Escherichia coli* strains

Table S8. Statistics and MOB genotypes of the 117 reconstructed plasmids

Table S9. Details of five isolates with complete genome sequences finished by Illumina and ONT sequencing

Supplemental Figures

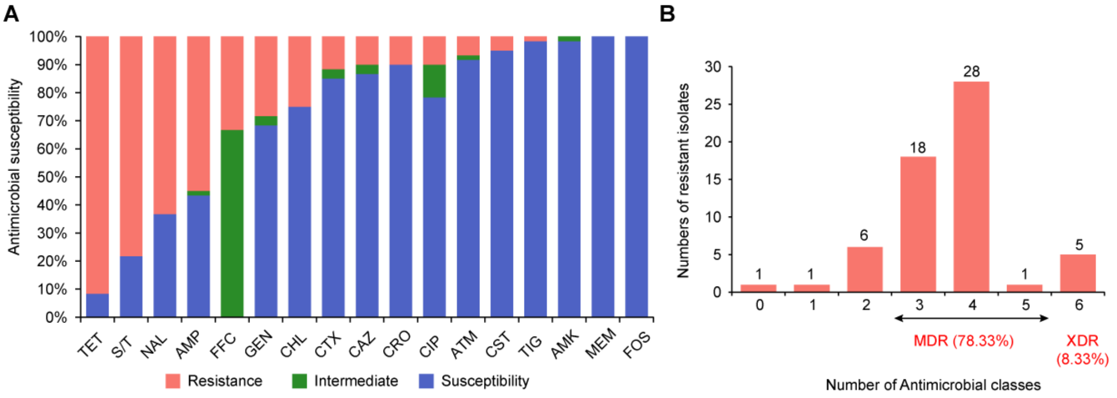

Figure S1. Antimicrobial resistance characteristics of 60 *Escherichia coli* strains isolated from this study

(A) The percentage of *E. coli* isolates resistant to each of the antibiotics tested;

(B) Numbers of *E. coli* isolates resistant to different antibiotic classes tested.

TET: tetracycline, S/T: sulfamethoxazole/trimethoprim, NAL: nalidixic acid, AMP: ampicillin, FFC: florfenicol, GEN: gentamicin, CHL: chloramphenicol, CTX: cefotaxime, CAZ: ceftazidime, CRO: ceftriaxone, CIP: ciprofloxacin, ATM: aztreonam, CST: colistin, TIG: tigecycline, AMK: amikacin, MEM: meropenem, FOS: Fosfomycin.

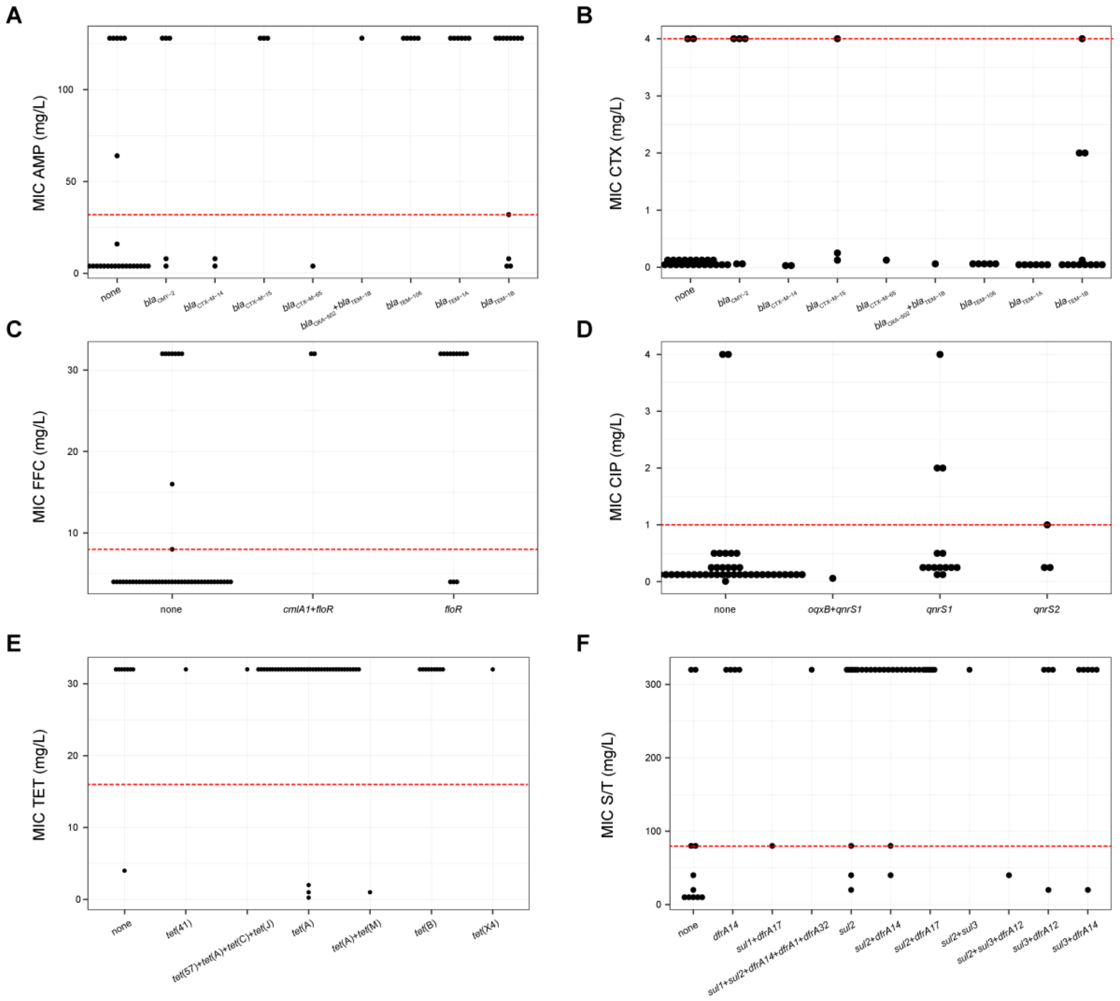

Figure S2. Association of AMR genotype and phenotype for first- and second-line antimicrobials

Genotype-phenotype association for ampicillin **(A)**, ceftriaxone **(B)**, florfenicol **(C)**, ciprofloxacin **(D)**, tetracycline **(E)**, sulfamethoxazole/trimethoprim **(F)**. Dots indicate the minimum inhibitory concentration (MIC) (y-axis) for each of 60 isolates harbouring a certain combination of genomic resistance markers (shown on the x- axis). The dashed line in red indicates the CLSI M100-S28 and VET01-S2 breakpoint for clinical resistance.

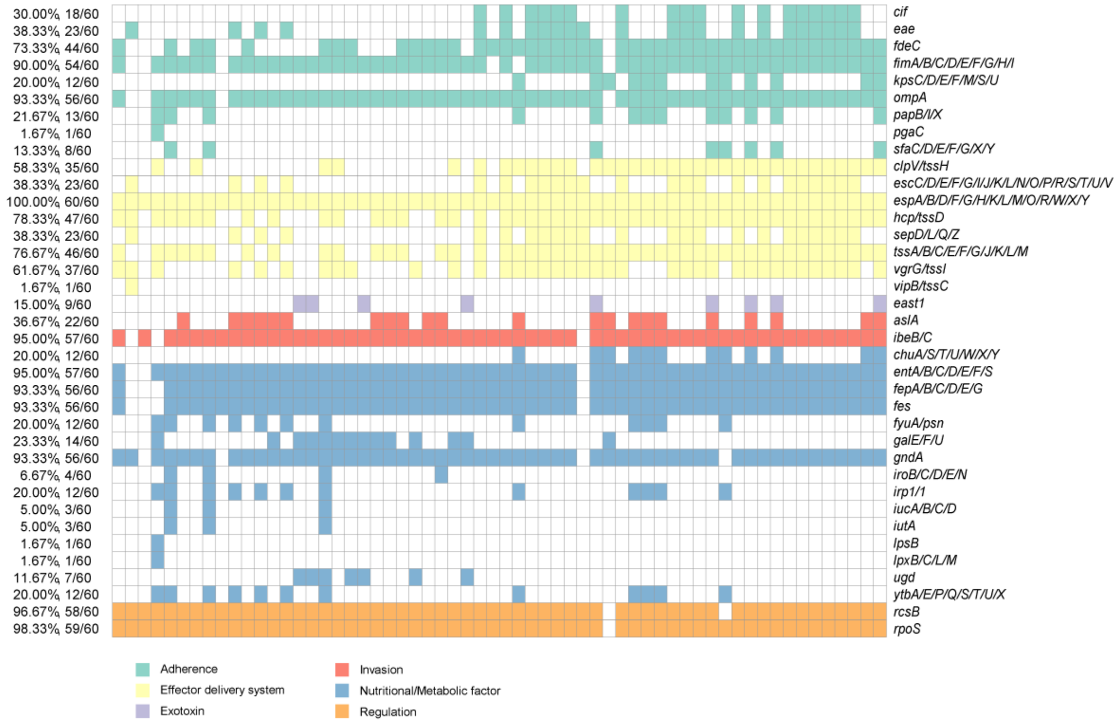

Figure S3. Virulence factors and genes in 60 *Escherichia coli* from pet cafés

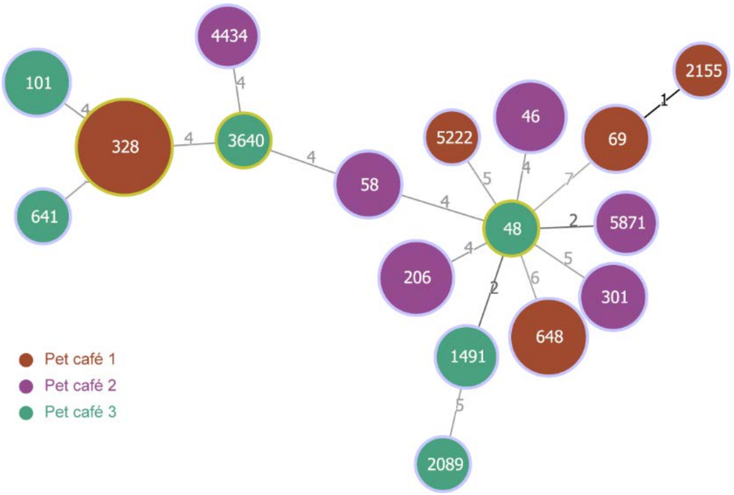

Figure S4. Minimum spanning trees of *Escherichia coli* by MLST type and gene allele profile from pet cafés
